# Biological Activity of 3-Hydroxypyridine Derivatives with Natural Components

**DOI:** 10.3390/ijms27198903

**Published:** 2026-10-07

**Authors:** Irina V. Zhigacheva, Elena M. Mil, Irina F. Rusina, Natalya I. Krikunova, Ludmila I. Matienko, Timur L. Veprintsev, Anastasia A. Albantova, Alexander N. Goloshchapov

**Affiliations:** 1 Emanuel Institute of Biochemical Physics, Russian Academy of Sciences, St. Kosygin, 4, Moscow 119334, Russia; elenamil2004@mail.ru (E.M.M.); kric@mail.ru (N.I.K.); matienko@sky.chph.ras.ru (L.I.M.); timlev@yandex.ru (T.L.V.); albantovaaa@mail.ru (A.A.A.); golan@sky.chph.ras.ru (A.N.G.); 2 N.N. Semenov Federal Research Center for Chemical Physics, Russian Academy of Sciences, St. Kosygin, 4, Moscow 119334, Russia; rusina939@mail.ru

**Keywords:** mitochondria, LPO (lipid peroxidation), fatty acids, autophagy, antioxidants, 3-hydroxypyridines

## Abstract

We investigated the antiradical properties of antioxidants: carnitinate 2-ethyl-6-methyl-3-hydroxypyridine (CAR-3-HP) and carboxychromate-2-ethyl-6-methyl-3-hydroxypyridine (CCR-3-HP), and also assessed the mitochondrial response to stress (hypoxia) by analyzing the intensity of lipid peroxidation, membrane fatty acid composition, and activation of autophagy markers LC3 and Beclin-1 using spectrofluorimetry, chromatography, and immunoblotting, respectively. The studied antioxidants exhibited high antiradical activity, which was reflected in their ability to inhibit lipid peroxidation. Five-day administration of antioxidants to mice at a dose of 2 × 10^−9^ mol/kg prevented the activation of lipid peroxidation in liver mitochondrial membranes under conditions of acute hypobaric hypoxia. The drugs also prevented a decrease in C_18_ and C_20_ fatty acid levels, and also activated autophagy. In both cases, CAR-3-HP was more effective than CCR-3-HP. This drug increased the lifespan of mice almost twice as effectively under conditions of various types of hypoxias.

## 1. Introduction

Under stress, an increase in the level of catecholamines in the blood leads to the release of Ca^2+^ from the vascular system into the cells. This causes it to accumulate in the mitochondria, leading to increased production of reactive oxygen species (ROS). Depending on the intensity of the stress, ROS can initiate adaptive reactions of the cell or provoke disturbances in its metabolism [1]. In this process, mitochondria play a dual role: they are both the site of formation of these intermediates and the target of their action. Excessive production of reactive oxygen species (ROS) provokes oxidative modification of protein thiol groups and intensification of lipid peroxidation processes, which ultimately leads to mitochondrial swelling and a decrease in their functional activity [2]. Moreover, overproduction of ROS can modulate the activity of redox-dependent signaling cascades, which initiates apoptotic mechanisms [3]. It is assumed that antioxidants, by reducing the mitochondrial ROS generation, will prevent these processes and increase the body’s resistance to stress.

Moreover, it is known that a number of antioxidants can induce autophagy [4], the activation of which leads to the elimination of damaged organelles, which increases the body’s resistance to oxidative stress. Furthermore, it has been established that autophagy is involved in regulating both the production and elimination of reactive oxygen species (ROS), coordinating the release and activation of the nuclear factor Nrf2 (nuclear factor erythroid 2-related factor 2) [5].

3-hydroxypyridine derivatives with antioxidant activity were selected as the object of the study. This compound serves as a key structural fragment in the synthesis of a variety of biologically active substances, including pharmaceuticals, antibiotics, and herbicides. The variety of biological effects characteristic of 3-hydroxypyridine derivatives determines the relevance of their study. Probably, the spectrum of biological activity observed in its derivatives are related to their involvement in the regulation of signaling pathways mediated by the transcription factor NF-kB [6]. The structural similarity of 3-hydroxypyridine (3-HP) derivatives to pyridoxine (vitamin B_6_) is also associated with their biological activity. Given the latter’s key role in carbohydrate, protein, and lipid metabolism, as well as its involvement in neurotransmitter synthesis and erythropoiesis, it can be assumed that structural analogs of 3-HP are capable of exerting comparable physiological effects on the body [7]. Moreover, 3-HP derivatives exhibit pronounced antioxidant activity due to their structural similarity to aromatic phenols. Being heterocyclic analogs of the latter, these compounds effectively neutralize free radicals, acting as physiological antioxidants [8]. Showing antioxidant properties, 3-hydroxypyridine derivatives can probably serve as adaptogens under various stressful conditions.

**Figure 1:**
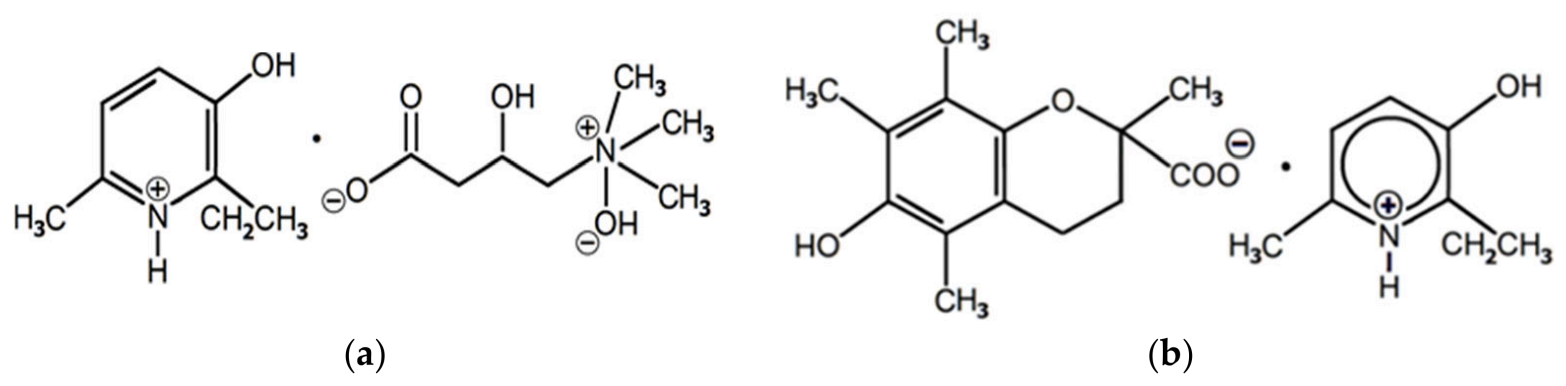
(**a**) CAR-3-HP, (**b**) CCR-3-HP.

Two derivatives of 3-HP were selected for the study (Figure 1): carnitinate 2-ethyl-6-methyl-3-hydroxypyridine (CAR-3-HP) and carboxychromate-2-ethyl-6-methyl-3-hydroxypyridine (CCR-3-HP):

L-carnitine, a component of CAR-3-HP, enhances energy metabolism by promoting the transmembrane transport of long-chain fatty acids into the mitochondrial matrix for subsequent β-oxidation. L-carnitine’s regulation of the CoASH/acetylCoA ratio helps optimize mitochondrial metabolism and suppress excessive lactic acidosis [9]. Among other things, L-carnitine modulates signaling pathways by activating the transcription factors Nrf2 and PPARα while inhibiting NF-κB. This mechanism leads to increased transcription of genes responsible for the synthesis of antioxidant enzymes [10].

In addition to 2-ethyl-6-methyl-3-hydroxypyridine, CCR-3-HP contains a synthetic analog of α-tocopherol, 6-hydroxy-2,5,7,8-tetramethylchroman-2-carboxylic acid (Trolox). This component significantly enhances the antioxidant potential of the drug: by effectively neutralizing peroxyl and alkoxyl radicals, Trolox significantly enhances the drug’s cytoprotective properties [11].

## 2. Results

### 2.1. Antiradical Activity of Carnitinate 2-Ethyl-6-Methyl-3-Hydroxypyridine (CAR-3-HP) and Carboxychromanate-2-Ethyl-6-Methyl-3-Hydroxypyridine (CCR-3-HP)

Across all the experiments presented, CAR-3-HP or CCR-3-HP were dissolved in double-distilled water.

**Figure 2:**
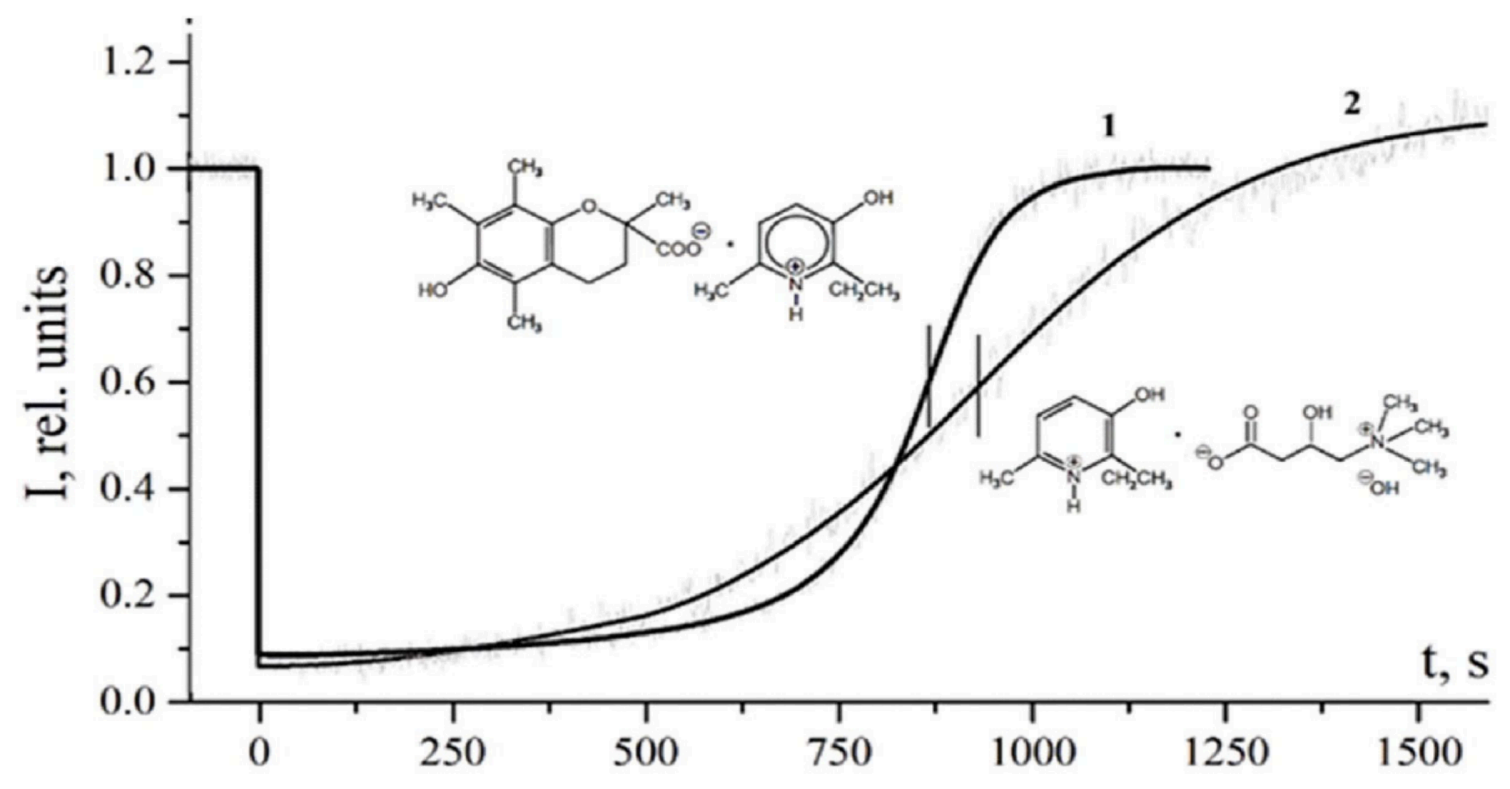
Time profiles of the chemiluminescence emission (intensity, I, in relative units) derived from the model reaction mixture of ethylbenzene (20% in chlorobenzene), which has been oxidized with the initiation rate Wi = 1.12·10^−8^ (M × s)^−1^ for CAR-3-HP (2) and Wi = 8.1·10^−8^ (M × s)^−1^ for CCR-3-HP (1) at 50 °C in the presence of 5 × 10^−3^ M of DBA used as the chemiluminescence enhancer upon addition of 9.77 × 10^−6^ M of the standard chain-breaking antioxidant chromane C_1_, CrC_1_. Concentration of CAR-3-HP was 5.2·10^−6^ M and concentration of CCR-3-HP was 1.87·10^−6^ M.

The antiradical activity of CAR-3-HP and CCR-3-HP was evaluated using chemiluminescence techniques, as detailed in [12]. A model reaction of ethylbenzene oxidation, initiated by azo-bisisobutyronitrile (AIBN) at 50 °C, was used as a test system. The experiment was conducted according to a previously published methodology [13]. During the oxidation of ethylbenzene, an excited state that promotes light emission occurs due to the reaction of disproportionation of two ethylbenzene peroxide (RO_2_***^·^***) radicals. This step in the chain oxidation process produces two electronically excited compounds—a triplet ketone (acetophenone) and singlet oxygen—both capable of emitting photons. However, light emitted either by an excited ketone (direct phosphorescence) or by an emission amplifier, a suitable luminophore, is usually detected. The luminophore is excited by nonradiative energy transfer from a triplet ketone. In this study, 9,10-dibromoanthracene (DBA) was used as such an enhancer. Adding appropriate antioxidants capable of chain termination and acting as inhibitors (InH) of the oxidative process results in quenching of the chemiluminescence. These inhibitors neutralize RO_2_**^·^** radicals. As InH (in this case, CAR-3-HP or CCR-3-HP) is gradually consumed, the concentration of RO_2_**·** radicals in a quasi-steady state is restored, which leads to a return of the chemiluminescence intensity level. Figure 2 shows the time profiles of chemiluminescence recorded after the introduction of CAR-3-HP or CCR-3-HP components into a chemiluminescent “cocktail” based on oxidizable ethylbenzene. To control the initiation rate (Wi) of free radical oxidation both before and after recording the time profiles of chemiluminescence in the presence of CAR-3-HP or CCR-3-HP, the standard antioxidant chroman C_1_ (CrC_1_), which is a synthetic analog of α-tocopherol, was used. At a known initial concentration of chromane C_1_, [CrC_1_]_0_, the reaction initiation rate is determined using Equation (1):

Wi = *f* [InH]_0_/τ_0.5_ = 2[CrC_1_]_0_/τ_0.5_ (1)

τ_0.5_ is the chemiluminescence induction period; *f* = 2 is the stoichiometric coefficient of CrC_1_, reflecting the number of peroxyl radicals (namely, two) absorbed per CrC_1_ molecule.

The kinetics of recovery of chemiluminescence intensity reflects the antiradical activity of the drug that terminates the chain, which is expressed by the rate constant (k_inh_) of the interaction of InH with RO_2_ [13] (Figure 2).

The k_inh_ parameter can be obtained from the maximum slope, (dI/dt)_max_, of the chemiluminescence intensity curve, I(t), (at the inflection point), using Equation (2), in which k_t_ is the rate constant for the disproportionation of RO_2_ (for ethylbenzene, k_t_ = 1.9 × 10^7^ (M × s)^−1^, at the terminal stage of the oxidation chain reaction:

(dI/dt)_max_ = 1/T = 0.237(k_inh_/(2 k_t_)^1/2^)Wi^1/2^ (2)

The stoichiometric coefficient (*f*), reflecting the number of radicals neutralized by one antioxidant molecule, serves as a quantitative indicator of its effectiveness. This value is calculated according to the following equation:

*f* [InH]_0_ = Wi × τ_0.5_ (3)

The antiradical activity of CAR-3-HP and CCR-3-HP, characterized by the inhibition constant (k_InH_) and the stoichiometric inhibition coefficient (*f*), are summarized in Table 1 to allow for comparison with known antioxidants.

Thus, the studied drugs have high antiradical activity and, possibly, they may influence the activation of lipid peroxidation.

### 2.2. Concentration Dependence of Lipid Peroxidation Inhibition by CAR-3-HP or CCR-3-HP

#### A Study on “Aging” Mitochondria

The ability of the studied drugs to inhibit lipid peroxidation was studied using a model of “aging” of mouse liver mitochondria. The mitochondrial fraction (2–3 mg of protein mass) was incubated in 0.5 mL of a buffer solution (65 mM KCl, 10 mM HEPES, 1 mM KH_2_PO_4_, pH 7.4) for 20–25 min at room temperature. The intensity of lipid peroxidation (LPO) was determined using fluorescence analysis according to the method [15]. Lipid extraction was performed from samples containing 3–5 mg of protein using a mixture of chloroform and methanol in a volume ratio of 2:1, with 10 mL of extraction mixture per milligram of mitochondrial protein.

**Figure 3:**
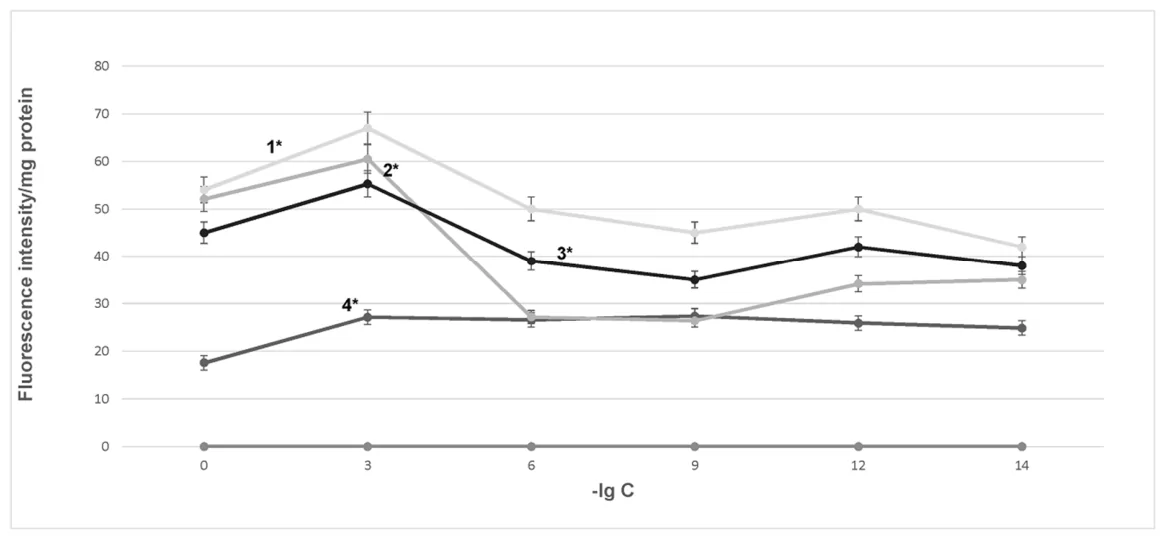
The effect of aging and CAR-3-HP or CCR-3-HP on the fluorescence intensity of lipid peroxidation products in mouse liver mitochondrial membranes. The coordinate axis shows the fluorescence intensity in arbitrary units/mg of protein; the abscissa axis corresponds to (-lg) concentrations of the studied antioxidants. 1—aging + dibunol (ionol) (Standard antioxidant); 2—aging + CAR-3-HP; 3—aging + CCR-3-HP; 4—control (without adding antioxidants). For each point, the number of technical repetitions was equal to 10. The curves were compared in pairs relative to the control or dibunol. Designations * refer to *p* < 0.01.

Mitochondrial aging caused activation of lipid peroxidation (LPO), which was manifested by increased fluorescence of its end products (Schiff bases). The fluorescence intensity of LPO products in mitochondrial membranes increased by 1.9–2.1 times (Figure 3).

Introduction of CAR-3-HP (Figure 3, curve 2) and CCR-3-HP (Figure 3, curve 3) into the mitochondrial incubation medium resulted in a concentration-dependent decrease in the lipid peroxidation intensity almost to that of the control values (curve 4), which, apparently, indicates the presence of protective properties in the drugs. It should be noted that the most effective concentrations for CAR-3-HP were 10^−6^–10^−12^ M and for CCR-3-HP - 10^−9^ M. It should be noted that CAR-3-HP and CCR-3-HP reduced the intensity of lipid peroxidation significantly more effectively than the standard antioxidant dibunol (ionol) (Figure 3, curve 1), whereas bidistilled water had no effect on fluorescence intensity. At a concentration of 10^−3^ M, the drugs exhibited a pro-oxidant effect, which can be explained by the fact that, when interacting with free radicals, antioxidants (in our case, CAR-3-HP or CCR-3-HP) transform into radicals with low reactivity. At high antioxidant concentrations, the level of antioxidant radicals rises to the point where they begin to attack target molecules and initiate new oxidation chains—thereby activating lipid peroxidation. The obtained data indicate the need to take into account the properties of the drugs to activate lipid peroxidation at this concentration.

### 2.3. Acute Hypobaric Hypoxia Triggers Lipid Peroxidation in the Membranes of Mouse Liver Mitochondria

The adaptogenic properties of the drugs were tested using the acute hypobaric hypoxia (AHH) model. Acute hypobaric hypoxia was induced in mice by placing them in a glass pressure chamber at a pressure of 230.40 mm Hg, simulating an altitude of 9000 m above sea level. This exposure lasted for 5 min. In the pre-exposure period, experimental mice were given daily intraperitoneal injections of CAR-3-HP or CCR-3-HP at a dose of 2 × 10^−9^ mol/kg for five days. Conversely, control mice received double-distilled water according to the same schedule and volume.

**Figure 4:**
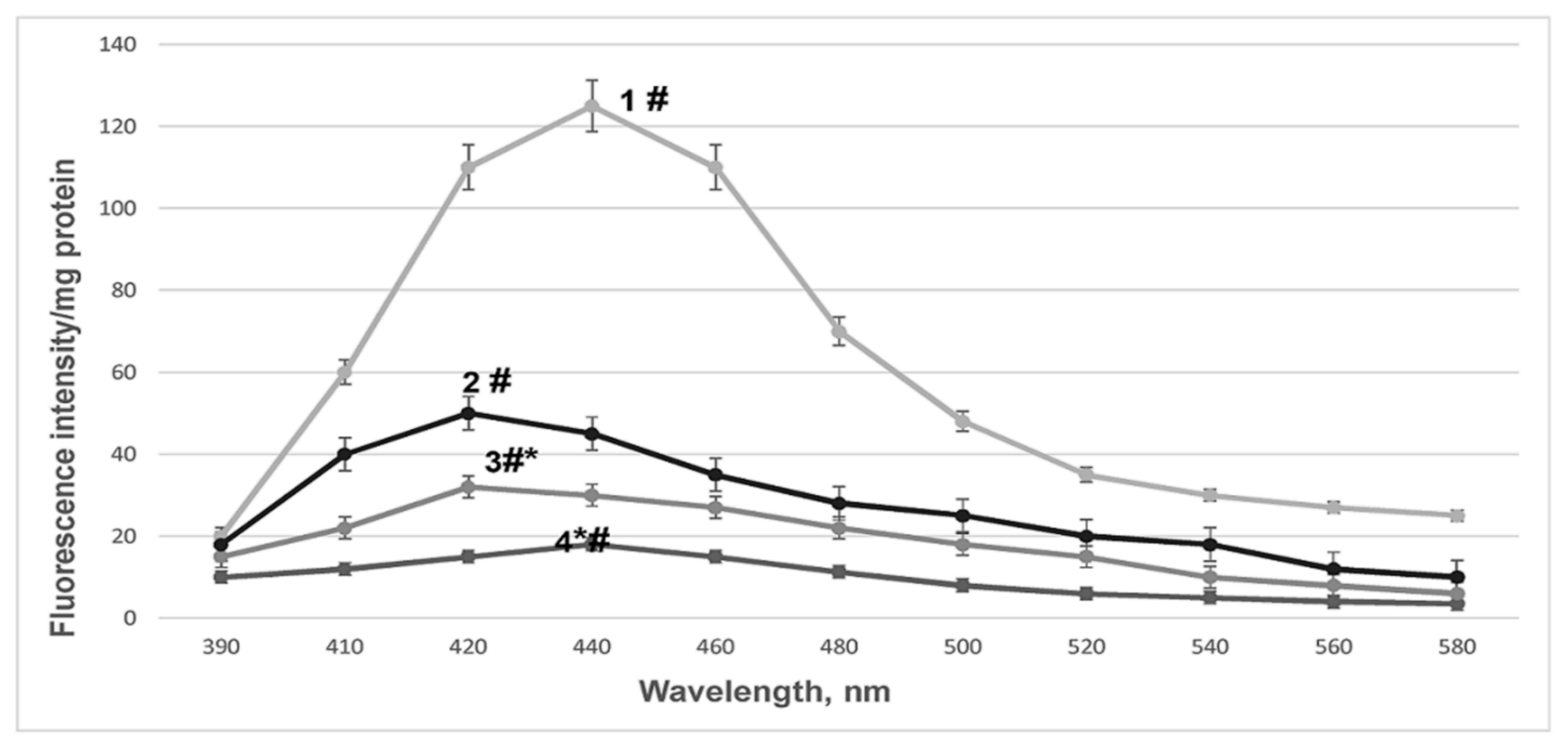
Effect of acute hypobaric hypoxia of Carnitinate 2-ethyl-6-methyl-3-hydroxypyridine (CAR-3-HP) or Carboxychromate 2-ethyl-6-methyl-3-hydroxypyridine (CCR-3-HP) on the fluorescence spectra of lipid peroxidation products in mouse liver mitochondrial membranes. Y-axis—fluorescence intensity in arbitrary units/mg of protein; the X-axis—emission wavelength. 1—AHH; 2—AHH + CCR-3-HP; 3—AHH + CAR-3-HP; 4—control (without using the studied antioxidants). For each spectrum, the number of technical replicates was 8, and the number of biological replicates was 3. The spectra were compared pairwise relative to the control. The value * corresponds to *p* < 0.05, and #—*p* < 0.01.

The choice of the AHH model is due to the activation of ROS generation by mitochondria under hypoxic conditions, resulting from the transition of complex I to an inactive state. Under conditions of acute hypoxia, this transition leads to acidification of the mitochondrial matrix and the release of Ca^2+^, ultimately resulting in superoxide generation at the level of complex III [16,17,18]. Excessive generation of ROS by mitochondria can be accompanied by activation of lipid peroxidation, caused by the interaction of these intermediates with polyunsaturated fatty acids that are part of the membranes’ lipid fraction. Indeed, AHH led to activation of lipid peroxidation. At the same time, a fivefold increase in the fluorescence intensity of lipid peroxidation products was observed. This effect is consistent with the results of studies conducted by M.S. Kislin and his group [17] (Figure 4).

Injection of antioxidants to mice prevented LPO activation: the fluorescence intensity of lipid peroxidation products approached the control level (Figure 4, curve 3).

However, Carnitinate 2-ethyl-6-methyl-3-hydroxypyridine carnitine (CAR-3-HP) was more effective, reducing the fluorescence intensity of LPO products by 15.7% (Figure 4, curve 3) compared to Carboxychromate 2-ethyl-6-methyl-3-hydroxypyridine (CCR-3-HP) (Figure 4, curve 2).

### 2.4. Fatty Acid Composition of Mouse Liver Mitochondrial Membranes After Administration of Carnitinate 2-Ethyl-6-Methyl-3-Hydroxypyridine Carboxychromanate 2-Ethyl-6-Methyl-3-Hydroxypyridine Under Conditions of Acute Hypobaric Hypoxia

The initiation of lipid peroxidation processes could likely have influenced the fatty acid (FA) composition of the lipid fraction of mitochondrial membranes. To investigate how lipid peroxidation impacts the fatty acid makeup of mouse mitochondrial membranes, a synchronized sacrifice of one mouse per group occurred at 10 am. This procedure was repeated for a total of 10 mice from each experimental cohort. The analysis of fatty acid composition in mouse liver mitochondrial membranes was performed using gas chromatography coupled with mass spectrometry.

Mitochondrial membrane lipids were first converted into fatty acid methyl esters (FAMEs). Fatty acid methyl esters (FAMEs) were generated from mitochondrial membrane lipids via acid methanolysis [19,20]. These FAMEs were then isolated with hexane, and the resulting hexane solutions subjected to analysis.

**Figure 5:**
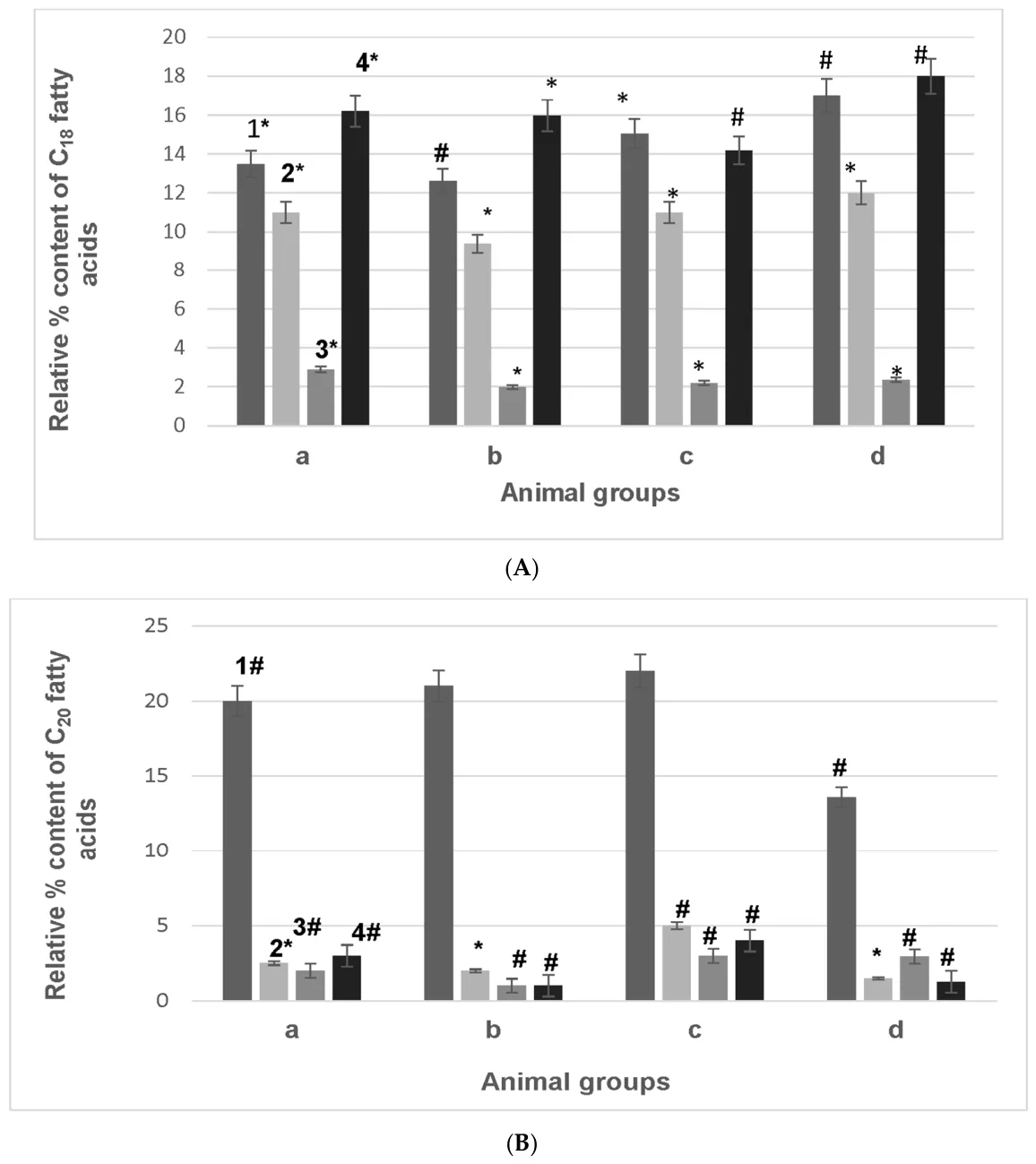
(**A**) The relative content of C_18_ fatty acids in mouse liver mitochondrial membranes under acute hypobaric hypoxia, with CAR-3-HP or CCR-3-HP and without injection. Animal groups: a—control; b—AHH; c—AHH + CAR-3-HP; d—AHH + CCR-3-HP. 1—18:2ω6; 2—18:1ω9; 3—18:1ω7; 4—18:0. For each group, the number of technical replicates was 6, and the number of biological replicates was 3. The relative content of fatty acids was compared in pairs relative to the control or relative to acute hypobaric hypoxia. The value * corresponds to *p* < 0.05, and #—*p* < 0.01. (**B**) The relative content of C_20_ fatty acids in mouse liver mitochondrial membranes under acute hypobaric hypoxia, with CAR-3-HP or CCR-3-HP and without injection. Animal groups: a—control; b—AHH; c—AHH + CAR-3-HP; d—AHH + CCR-3-HP. 1—20:4ω6; 2—20:3ω3; 3—20:2ω6; 4—20:1ω9. For each group, the number of technical replicates was 6, and the number of biological replicates was 3. The relative content of fatty acids was compared in pairs relative to the control or relative to acute hypobaric hypoxia. The value * corresponds to *p* < 0.05, and #—*p* < 0.01.

Acute hypobaric hypoxia caused changes in the content of C_18_ and C_20_ fatty acids (FA) (Figure 5A,B).

Thus, the content of 18:2ω6 (Figure 5A, column 1) decreased by 7%, and the content of 18:1ω9 by 15% (Figure 5A, column 2). The stearic acid pool remained almost unchanged (Figure 5A, column 4). At the same time, the double bond index (DBI) of C_18_ FA, calculated using the formula: DBI = Σ(P_j_ × n)/100, where P_j_ is the fatty acid content (relative %) and n is the number of double bonds in each acid, decreased from 0.83 ± 0.01 to 0.53 ± 0.01. The injection of antioxidants to mice prevented changes in the content of C_18_ unsaturated FAs under conditions of acute hypobaric hypoxia. At the same time, there was an increase in the content of 18:2ω6 in mitochondrial membranes by 11% (CAR-3-HP) and 26% (CCR-3-HP) compared to the control.

Changes in the content of C_20_ FA were also observed (Figure 5B). The content of 20:3ω3 in mitochondrial membranes under AHH conditions decreased by 13%, 20:2ω6 by 80%, and 20:1ω9 by 33% (columns 2, 3 and 4, respectively). The ratio between FA—precursors and inhibitors of eicosanoid synthesis 20:4ω6/22:6ω3—decreased from 2.22 ± 0.12 to 1.43 ± 0.10, which indicated a decrease in metabolism in the eicosanoid cycle. Since prostaglandins perform many regulatory functions, such as modulating enzymatic activity, influencing hormone synthesis and their effects on various organs and tissues [21], a decrease in the level of their precursors, probably, as well as a decrease in the content of linoleic acid, could reduce the body’s resistance to stress. Administration of CAR-3-HP prevented these changes. Furthermore, 20:4ω6 levels increased by 10% compared to the control group. Concurrently, 20:3ω3 and 20:2ω6 levels also increased by 80% and 50%. Regarding CCR-3-HP, although administration of this drug increased C_20_ fatty acid levels, it did not restore them to control values.

### 2.5. Studies of the Ability of CAR-3-HP or CCR-3-HP to Activate Autophagy

Autophagy is known to be the main cellular defense against oxidative stress or related conditions that cause accumulation of damaged proteins or organelles [22]. In this context, the activation of autophagy is probably accompanied by an increase in the body’s resistance to oxidative stress [23]. It occurs in response to nutrient deficiency, starvation, the presence of damaged cellular structures, partially denatured protein molecules and their clusters, as well as oxidative or toxic stress [24,25].

Morphological markers indicating the activation of autophagy include the formation of vacuoles (autophagosomes), as well as changes in the size of organelles: a decrease in the volume of mitochondria and the area of the endoplasmic reticulum with a simultaneous increase in the Golgi apparatus [26].

One approach to studying autophagy is the use of antibodies specific to the LC3 and Beclin-1 proteins. The LC3 protein is unique in that it is present in both the inner and outer membranes of autophagosomes. During Western blotting, the LC3 protein family is usually detected as multiple bands corresponding to the LC3A, LC3B, LC3C, as well as LC3-I and LC3-II isoforms. It has previously been established that the autophagosomal proteins LC3A, LC3B, and LC3C differ in their expression levels and subcellular localization in cancer cell lines [27].

The key regulator of the formation and development of autophagosomes is the Beclin-1 protein, which does this by interacting with other protein structures within the PI3KC3-C1 complex, which plays a decisive role in initiating the biogenesis of autophagosomes [28,29].

**Figure 6:**
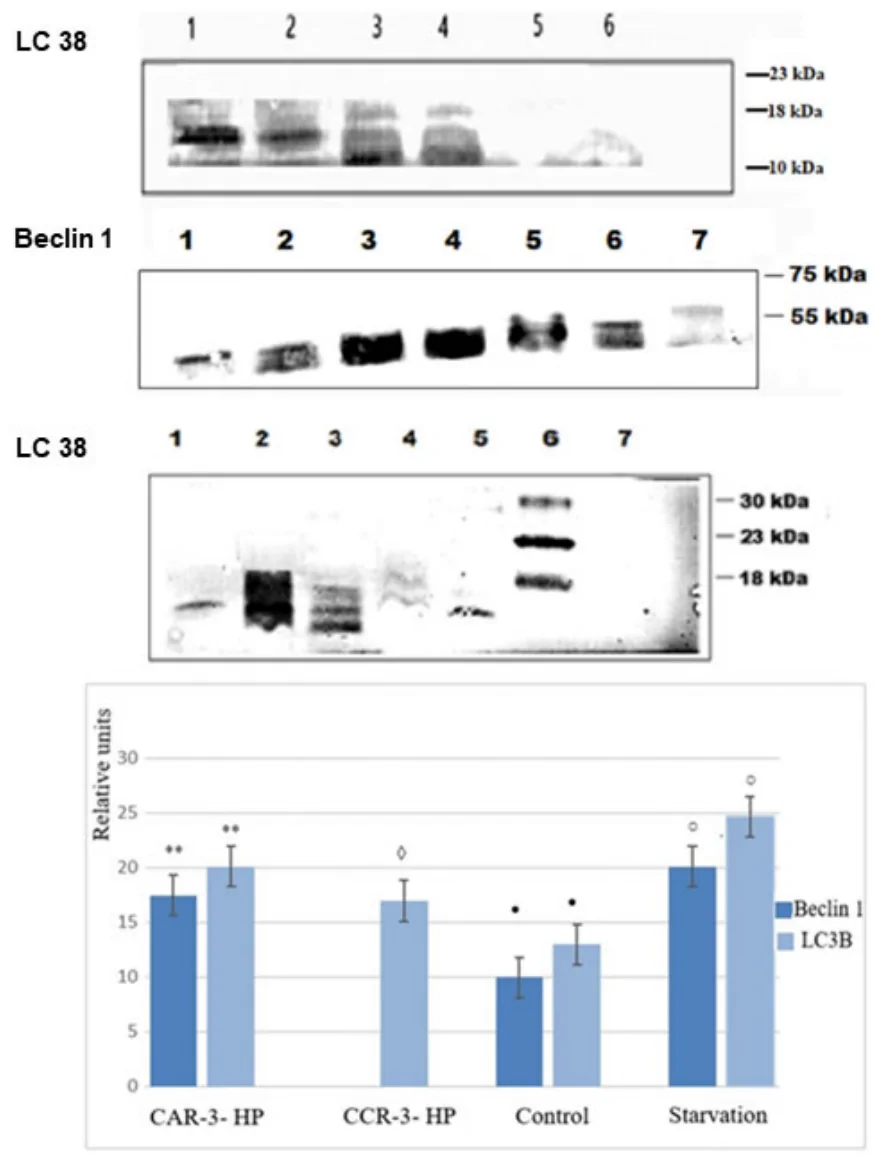
Western blotting (**A**) of LC3B and (**B**) Beclin1 proteins: from left to right, preparations in columns: 1,2—Starvation of mice, 3,4—CAR-3-HP, 5,6—Control, 7—Protein marker; (**C**) of LC3B: 1,4,5—Control, 2—CAR-3-HP, 3—CCR-3-HP, 6—Protein marker. Diagram (**D**) of the LC3B and Beclin1 protein content in liver cells after addition of the studied antioxidants to mice and during starvation: 1—CAR-3-HP; 2—CCR-3-HP; 3—Control; 4—Starvation. For each group, the number of technical replicates was 6, and the number of biological replicates was 3. The relative content of LC3B and Beclin1 protein content in liver cells were compared in pairs relative to the control or relative to starvation. ** *p* < 0.01; ◊ *p* < 0.05, ● *p* < 0.001, ◦ *p* < 0.001.

The effects of antioxidants (CAR-3-HP and CCR-3-HP) at 10^−5^ mol/kg and 2-day starvation on LC3B and Beclin1 protein levels in mouse liver cells were studied. Western blot data using antibodies to these proteins are shown, along with corresponding densitograms for assessing their levels in liver cells. Figure 6 demonstrates stained bands on a nitrocellulose membrane, obtained after transfer from a 10% gel, corresponding to LC3B and Beclin-1 proteins.

Western blot analysis and protein densitogram data indicate that CAR-3-HP (with 3-day administration) is effective in activating the autophagy biomarker LC3B in mice. Moreover, the LC3B protein level was 60% higher than its control level. At the same time, in the “starvation” group, LC3B levels were significantly higher than in the control group (two days of starvation) This may indicate that in the liver cells of mice from these groups activation of markers was associated with autophagy, which is consistent with the modulation of this pathway. It has previously been shown that its various complexes with proteins such as LC3/ATG, as well as with RNA, play a crucial role in autophagy, transferring various cytoplasmic materials into lysosomes [30].

In the 10^−5^ mol/kg CAR-3-HP groups and during “starvation” (Figure 6A,B), a close increase in the content of Beclin-1 protein, which regulates the formation and maturation of autophagosomes, was also found in relation to the control.

The mechanism underlying the differences in the effects of these antioxidants on the activation of autophagy biomarkers has yet to be elucidated; however, the data obtained are consistent with the fact that CAR-3-HP is more effective in reducing the intensity of lipid peroxidation compared to CCR-3-HP.

### 2.6. Anti-Stress Properties of CAR-3-HP and CCR-3-HP

Modification of the physicochemical characteristics of mitochondrial membranes may have affected their functional activity, which in turn could determine the body’s resistance to stress. The use of drugs in mice demonstrated their protective effect under stress conditions. Thus, the intraperitoneal injection of 2 × 10^−9^ mol/kg CAR-3-HP to mice increased the lifespan by 3.2–4.3 times and increased survival of mice by 12–35% under conditions of various types of hypoxia. Moreover, the drug increased dynamic performance by 3.5 times. It should be noted that the injection of 2 × 10^−9^ mol/kg CCR-3-HP increased the lifespan by 1.5–1.9 times and increased survival of mice by 9–22% under these conditions (Tables S1 and S2 respectively, Supplementary Materials).

## 3. Discussion

The obtained results suggest that the studied antioxidants, probably, can serve as adaptogens due to their high efficiency in inhibiting free radicals and the intensity of lipid peroxidation [31]. Some antioxidants have previously been shown to have the ability to induce autophagy [4]. Indeed, CAR-3-HP and CCR-3-HP activate in the mouse liver cells activation of markers associated with autophagy. Therefore, it can be hypothesized that the induction of autophagy flux is also associated with increased organismal resistance to oxidative stress [5].

2-ethyl-6-methyl-3-hydroxypyridine, which is a part of the preparations, exhibits the ability to react with peroxide radicals present in lipids, peptides and protein structures. As a 3-hydroxypyridine derivative, this compound enhances the function of antioxidant enzymes, including catalase and superoxide dismutase, etc. [6,7].

Modifying the 3-HP molecule with different substituents conferred new properties upon the studied drugs, supplementing their inherent antioxidant activity. Thus, the introduction of L-carnitine resulted in a reduction in mitochondrial ROS generation due to L-carnitine binding to Fe^2+^ and Cu^2+^ ions in the enzyme’s active sites. In addition, L-carnitine is capable of inducing and modulating autophagy flux [32]. It has been proven that L-carnitine is able to activate mitophagy by inducing autophagy through PPARy [33]. L-Carnitine promotes the activation of the Nrf2 and PPARα signaling pathways while inhibiting NF-κB. This process leads to increased expression of genes encoding antioxidant enzymes. By stimulating the synthesis of protective molecules such as sirtuins, thioredoxins, heat shock proteins, and low-molecular-weight antioxidants, L-carnitine demonstrates its adaptogenic properties [34].

The inclusion of a Trolox fragment in the structure of the 2-ethyl-6-methyl-3-hydroxypyridine molecule, which is a 6-hydroxy-2,5,7,8-tetramethylchroman-2-carboxylic acid, plays a key role in increasing the antiradical and antioxidant activity of the molecule due to its high efficiency in neutralizing peroxyl and alkoxyl radicals [11]. At low concentrations, Trolox effectively reduces the intensity of lipid peroxidation [35]. However, when interacting with free metal ions, its properties change to pro-oxidant ones. As the concentration increases, Trolox begins to actively stimulate lipid peroxidation, oxidize glutathione (GSH), and exhibit cytotoxicity [36,37].

Trolox, as well as L-carnitine, can activate autophagy flux. This reveals a synergistic mechanism through which antioxidant and autophagic reactions interact, increasing the resistance of cells and the entire body to stress [38].

To date, a series of new compounds have been developed and synthesized, representing ethers and amides of Trolox. These newly obtained drugs demonstrated pronounced anti-inflammatory, antiradical, and antioxidant activity [39].

It is important to emphasize that the protective effect of the studied antioxidants is observed at lower concentrations than that of the mitochondria-targeted antioxidants MitoQ and SkQ [40]. The advantage of mitochondria-targeted antioxidants lies in their ability to concentrate in the mitochondrial matrix, which is determined by the membrane potential. This targeted accumulation allows them effectively suppress the formation of reactive oxygen species (ROS) by mitochondria [41]. However, their disadvantage is their inherent instability and limited bioavailability. In addition, damaged mitochondria demonstrate a reduced ability to absorb these drugs due to a decrease in membrane potential [42]. In contrast, CCR-3-HP and CAR-3-HP are released as stable and water-soluble substances, which is a clear advantage.

We found that CCR-3-HP and CAR-3-HP increased the animals’ resistance to hypoxia. By preventing lipid peroxidation, they not only protected C_18_ unsaturated fatty acids from oxidative damage but also increased primarily the content of linoleic acid (one of the main components of cardiolipin) [43] in mitochondrial membranes which likely supported optimal functioning of the mitochondrial respiratory chain [44,45] and preserved cellular energy metabolism. In addition, the studied drugs affected the content of arachidonic acid, a precursor of prostaglandins, leukotrienes, and some eicosanoids. An increase in arachidonic acid content, as well as an increase in linoleic acid content in mitochondrial membranes, may have increased the body’s resistance to stressors.

A comparative analysis showed that Carboxychromate 2-ethyl-6-methyl-3-hydroxypyridine (CCR-3-HP) is inferior to Carnitinate 2-ethyl-6-methyl-3-hydroxypyridine (CAR-3-HP) in terms of adaptogenic effect. This difference is likely due to the specific mechanism of action: CCR-3-HP is characterized by a predominance of antiradical activity over antioxidant activity, while CAR-3-HP exhibits high antiradical and antioxidant properties.

## 4. Materials and Methods

### 4.1. Materials

Carnitinate 2-ethyl-6-methyl-3-hydroxypyridine (CAR-3-HP) and Carboxychromate-2-ethyl-6-methyl-3-hydroxypyridine (CCR-3-HP) were first synthesized by researchers at the N.N. Semenov Institute of Chemical Physics of the Russian Academy of Sciences and the N.M. Emanuel Institute of Biochemical Physics of the Russian Academy of Sciences. The drugs are patented: CAR-3-HP (Russian Patent No. 2023117835), CCR-3-HP (Russian Patent No. 2025111343). The experiment used reagents of the following companies:

Methanol, chloroform (Merck, Darmstadt, Germany), sucrose, Tris, (Sigma, St. Louis, MO, USA), HEPES (MP Biomedicals, Eschwege, Germany), potassium carbonate, (Merck, Darmstadt, Germany), hexane (Panreac, Barcelona, Spain), acetyl chloride (Acros Organics, Geel, Belgium), primary antibodies against Beclin-1 and LC3B (TransGenBiotech, Beijing, China), secondary antibodies against mouse IgG conjugated with horseradish h peroxidase (HS201-01; ProteinFind^®^ GoatAnti-MouseIgG (H+ L), TransGenBiotech, Beijing, China), DAB (Biotium, San Francisco, CA, USA).

### 4.2. Fluorescence Measurements

This technique is aimed at quantitatively assessing the fluorescence of Schiff bases—products of the interaction of protein amino groups with aldehydes (malondialdehyde and 4-hydroxy-2,3-nonenal), which act as markers of lipid peroxidation (LPO). Spectra were recorded using a FluoroMax spectrofluorimeter (Horiba Yvon GmbH, Oberursel, Taunus, Germany) at an excitation wavelength of 360 nm and an emission range of 420–540 nm. Lipids were pre-extracted from mitochondrial fractions (containing 3–5 mg of protein) using a chloroform–methanol mixture in a 2:1 (volume) ratio.

### 4.3. Experiments with Animals and Biomaterials

Male Balb/c mice, aged three months and weighing 22–25 g, were selected for the experiments, with each group containing 10–15 individuals. CAR-3-HP or CCR-3-HP were administered intraperitoneally at 2 × 10^−9^ mol/kg daily for five days or for 3 days at a dose of 10^−5^ mol/kg in experiments studying the autophagy flux.

Doses were prepared by dissolving CAR-3-HP or CCR-3-HP in double-distilled water, with each mouse receiving 0.2 mL. The final CAR-3-HP or CCR-3-HP injection were given 45 min before stress exposure. All mice were fed a standard diet. A control group received double-distilled water in the same volume as the CAR-3-HP or CCR-3-HP solutions to account for solvent effects. This confirmed that double-distilled water did not impact mitochondrial function, ensuring observed effects were specific to CAR-3-HP or CCR-3-HP.

The study was conducted in accordance with the principles of biomedical ethics set out in the Helsinki Declaration of 1964 and subsequent amendments thereto (European Convention for the Protection of Vertebrates Used for Experimental and Other Scientific Purposes (ETS No. 123) [46], and was approved by the Ethics Committee of the Emanuel Institute of Biophysical Chemistry of the Russian Academy of Sciences (Moscow, Protocol No. 48, 12 December 2024).

Mitochondria were isolated from the mouse liver tissue using differential centrifugation [45]. The initial step involved a 10 min centrifugation at 600 g. The supernatant was then subjected to a second centrifugation for 10 min at 9000 g. The resulting pellet containing mitochondria was then resuspended in a buffer consisting of 0.25 M sucrose and 10 mM HEPES at pH 7.4. The isolated mitochondria, with a protein concentration of 2–3 mg, were then subjected to “aging,” which involved incubating them for 20–25 min at room temperature in 0.5 mL of a medium containing 65 mM KCl, 10 mM HEPES, and 1 mM KH_2_PO_4_ (pH 7.4).

The fatty acid profile of liver mitochondrial membranes was investigated using gas chromatography (GCh) and mass spectrometry (MS). Mitochondrial membrane lipids were first converted to fatty acid methyl esters (FAMEs) via acidic methanolysis [19,20]. Following hexane extraction, the FAME solutions were subjected to GCh analysis. For quantitative analysis of the FAMEs, a Kristall 2000 M gas chromatograph equipped with a flame-ionization detector and a DB-1 quartz capillary column (60 m × 0.32 mm, 0.25 µm phase film) from J&W Scientific was utilized. The chromatographic separation involved a temperature gradient, starting at 120 °C and rising to 270 °C at a speed of 4 °C per minute. The helium carrier gas flow rate was set to 2.0 mL/min with a split ratio of 1:40 at the column inlet. A 2 μL volume of the hexane extract was injected for each sample. FAME quantities were calculated as the ratio of individual peak areas to the sum of all identified FAME peak areas [47]. Triplicate analyses demonstrated a relative standard deviation of less than 5% for average peak areas. FAME identification was achieved by MS, performed after GC separation under identical conditions using a Hewlett Packard 6890 instrument. Mass spectra were acquired via electron impact ionization at 70 eV, with a scan rate of one cycle per mass decade across the 40–400 Dalton range.

Autophagy biomarkers in the liver homogenate were assessed via Western blotting. Primary antibodies against Beclin-1 and LC3B (TransGenBiotech, Beijing, China) were used, followed by detection with horseradish peroxidase-conjugated secondary antibodies against mouse IgG (HS201-01; ProteinFind^®^ GoatAnti-MouseIgG (H+ L), TransGenBiotech, Beijing, China). Protein bands, transferred to nitrocellulose membranes, were visualized using DAB staining for peroxidase activity (Biotium, San Francisco, CA, USA). Band intensity was subsequently measured using the BMP scale program (BMP Imige Editor download/Sourceforge.net; http://aourceforge.net/projects/bmp (accessed on November 26, 2010) editor.

The protective activity of the CAR-3-HP and CCR-3-HP was studied in Balb/c mice using models of acute hypobaric hypoxia and dynamic performance.

To simulate acute hypobaric hypoxia, mice were placed in a hyperbaric chamber. The chamber’s atmosphere was reduced to 230.40 mm Hg, simulating an altitude of 9000 m. The first minute of the simulation involved a rapid decrease in pressure to 405 mm Hg, corresponding to an altitude of 5000 m. Each subsequent minute saw a further reduction in pressure, simulating an additional 1000 m of ascent. The mice were maintained at the simulated 9000 m altitude for a duration of 5 min.

The dynamic performance of mice was determined in a forced swimming test with a load of 2% of body weight in water at room temperature until the animals were tired [48].

Statistical analysis was performed using the Student’s *t*-test. The obtained data were compared pairwise in relation to the control or stress exposure. Data are expressed as means ± standard deviations, with statistical significance defined as *p* ≤ 0.05. Calculations and data processing were conducted in Microsoft Excel and Sigma Plot 10.

## 5. Conclusions

The adaptogenic effect of CAR-3-HP and CCR-3-HP is probably related to their antiradical properties and the ability to inhibit lipid peroxidation. By preventing the oxidation of C_18_ and C_20_ unsaturated fatty acids, mainly linoleic acid, a key component of cardiolipin, and preventing disorders in the eicosanoid cycle, these drugs preserve the mitochondrial functional activity by supporting the energy metabolism of the cell, which may underlie the body’s resistance to stress. Furthermore, the drugs stimulate the production of autophagy biomarkers, LC3 and Beclin-1, which are associated with autophagy, suggesting modulation of this process and increased stress resistance.

## Figures and Tables

**Table 1:** Comparative antiradical activity of inhibitors (k_InH_, *f*).

Antioxidant	k_InH_ × 10^4^ (Ms)^−1^	*f*	Reference
α-Tocopherol	300	1.75	[14]
CAR-3-HP	23.0	2.0	-
CCR-3-HP	85.4–86.9	3.4–3.8	-
Mexidol	4.7	1.9	[13]
Chroman CrC_1_	452	2.0	[13]

## Data Availability

All original research findings are detailed within this article and its Supplementary Materials. Please address any follow-up questions to the designated corresponding author.

## Supplementary Materials

The following supporting information can be downloaded at: https://www.mdpi.com/article/10.3390/ijms27198903/s1.

## Abbreviations

The following abbreviations are used in this manuscript:

CAR-3-HP	Carnitinate 2-ethyl-6-methyl-3-hydroxypyridine
CCR-3-HP	Carboxychromate-2-ethyl-6-methyl-3-hydroxypyridine
3-HP	3-Hydroxypyridine
LPO	lipid peroxidation
ROS	reactive oxygen species
FAMEs	fatty acid methyl esters

## References

[1] Zorov D.B., Juhaszova M., Sollott S.J. (2014). Mitochondrial Reactive Oxygen Species (ROS) and ROS-Induced ROS Release. Physiol. Rev..

[2] Endale H.T., Tesfaye W., Mengstie T.A. (2023). ROS induced lipid peroxidation and their role in ferroptosis. Front. Cell Dev. Biol..

[3] Villalpando-Rodriguez G.E., Gibson S.B. (2021). Reactive Oxygen Species (ROS) Regulates Different Types of Cell Death by Acting as a Rheostat. Oxidative Med. Cell. Longev..

[4] Liu W., Hu M.D., Cao F., Jin S. (2025). Polyphenols and exercise in autophagy regulation: Potential benefits for cancer management and healthspan. Front. Nutr..

[5] Gouda N.A., Zhakupova A., Abdelaal A.M., Ahmad F., Elkamhawy A. (2025). The interplay involving oxidative stress and autophagy: Mechanisms, implications, and therapeutic opportunities. Exp. Mol. Pathol..

[6] Skachilova S.Y., Ragulina V.A., Kostina D.A., Burda Y.E. (2017). Test system for evaluation of the influence of the biological activity of substances on the signal system of NF-κB: Focus on the derivatives of 3- hydroxypyridine derivatives of 3- hydroxypyridine. Res. Result Pharmacol. Clin. Pharmacol..

[7] Dewangan S., Bhatia A.K., Verma C., Verma D.K. (2023). Vitamins and metabolites. Handbook of Biomolecules Fundamentals, Properties and Applications.

[8] Danilenko A.P., Trunov K.S., Pokrovsky M.V., Danilenko L.M., Korokin M.V., Gudyrev O.S., Khentov A.A., Masalytina N.P., Tatarenkova I.A., Cherednichenko A.V. (2023). Protective Role 3-Oxypyridine Derivations in Steroid-Induced Osteoporosis in Rats Associated with Reduction of oxidative Stress and Restoration of Nitric Oxide Formation. Pharm. Pharmacol..

[9] Gnoni A., Longo S., Gnoni G.V., Giudetti A.M. (2020). Carnitine in Human Muscle Bioenergetics: Can Carnitine Supplementation Improve Physical Exercise?. Molecules.

[10] Xiang F., Zhang Z., Xie J., Xiong S., Yang C., Liao D., Xia B., Lin L. (2025). Comprehensive review of the expanding roles of the carnitine pool in metabolic physiology: Beyond fatty acid oxidation. J. Transl. Med..

[11] Alberto M.E., Russo N., Grand A., Galano A. (2013). A physicochemical examination of the free radical scavenging activity of Trolox: Mechanism, kinetics and influence of the environment. Phys. Chem. Chem. Phys..

[12] Rusina I.F., Veprintsev T.L., Vasil’ev R.F. (2022). Antioxidant activity of diatomic phenols. Russ. J. Phys. Chem. B..

[13] Rusina I.F., Karpukhin O.N., Kasaikina O.T. (2013). Chemiluminescent methods for studying inhibited oxidation. Russ. J. Phys. Chem. B..

[14] Culbertson S.M., Antunes F., Havrilla C.M., Milne G.L., Porter N.A. (2002). Determination of the alpha-tocopherol inhibition rate constant for peroxidation in low-density lipoprotein. Chem. Res. Toxicol..

[15] Fletcher B.I., Dillard C.D., Tappel A.L. (1973). Measurement of fluorescent lipid peroxidation products in biological systems and tissues. Anal. Biochem..

[16] Wang H., Song T.-Y., Reyes-García J., Wang Y.-X. (2024). Hypoxia-Induced Mitochondrial ROS and Function in Pulmonary Arterial Endothelial Cells. Cells.

[17] Kislin M.S., Tiul’kova E.I., Samoĭlov M.O. (2011). Dynamics of lipid peroxidation of membranes in cells and mitochondrial fraction of neocortex in non- and preconditioned rats after severe hypobaric hypoxia. Zh. Evol. Biokhim. Fiziol..

[18] Dudylina A.L., Ivanova M.V., Shumaev K.B., Ruuge E.K. (2016). The generation of superoxide radicals by complex III in heart mitochondria and the antioxidant effect of dinitrosyl iron complexes at different partial pressures of oxygen. Biophysics.

[19] Carreau J.P., Dubacq J.P. (1979). Adaptation of macroscal method to the microscale for fatty acid methyl transesterification of biological lipid extracts. J. Chromatogr. A.

[20] Wang Y., Sunwoo H., Cherian G., Sim J.S. (2000). Fatty acid determination in chicken egg yolk: A comparison of different methods. Poult. Sci..

[21] Kushwaha V., Agrawal P., Vekaria H., Pathak B. (2023). Prostaglandins: An overview. Eur. J. Pharm. Med. Res..

[22] Ornatowski W., Lu Q., Yegambaram M., Garcia A.E., Zemskov E.A., Maltepe E., Fineman J.R., Wang T., Black S.M. (2020). Complex interplay between autophagy and oxidative stress in the development of pulmonary disease. Redox Biol..

[23] Rossin D., Perrelli M.-G., Lo Iacono M., Rastaldo R., Giachino C. (2025). Dynamic Interplay Between Autophagy and Oxidative Stress in Stem Cells: Implications for Regenerative Medicine. Antioxidants.

[24] Filomeni G., De Zio D., Cecconi F. (2015). Oxidative stress and autophagy: The clash between damage and metabolic needs. Cell Death Differ..

[25] Kroemer G., Mariño G., Levine B. (2010). Kalugina, Autophagy and the integrated stress response. Mol. Cell..

[26] Chang Y.-F., Li J.-J., Liu T., Wei C.-Q., Ma L.-W., Nikolenko V.N., Chang W.-L. (2024). Morphological and biochemical characteristics associated with autophagy in gastrointestinal diseases. World J. Gastroenterol..

[27] Koukourakis M.I., Kalamida D., Giatromanolaki A., Zois C.E., Sivridis E., Pouliliou S., Mitrakas A., Gatter K.C., Harris A.L. (2015). Autophagosome Proteins LC3A, LC3B and LC3C Have Distinct Subcellular Distribution Kinetics and Expression in Cancer Cell Lines. PLoS ONE.

[28] Cao Z., Tian K., Ran Y., Zhou H., Zhou L., Ding Y., Tang X. (2024). Beclin-1: A therapeutic target at the intersection of auto-phagy, immunotherapy, and cancer treatment. Front. Immunol..

[29] Chan W.W.R., Chow J., Chau D.D.-L., Zhai Y., Lau K.-F. (2025). Beclin 1-Mediated Autophagy Is Potentiated by an Interaction with the Neuronal Adaptor FE65. Biology.

[30] Hwang H.J., Ha H., Lee B.S., Kim B.H., Song H.K., Kim Y.K. (2022). LC3B is an RNA-binding protein to trigger rapid mRNA degradation during autophagy. Nat. Commun..

[31] Zhigacheva I.V., Rusina I.F., Krikunova N.I., Goloschapov A.N., Veprintsev T.L., Yablonskaya O.I., Trofimov A.V. (2023). Resveratrol and 2-Ethyl-6-Methyl-3-Hydroxypiridine N-Acetyl Cysteinate as Protecting Agents upon the Stress Exposure. Int. J. Mol. Sci..

[32] Khedr N.F., Werida R.H. (2022). L-Carnitine modulates autophagy, oxidative stress and inflammation in trazodone induced testicular toxicity. Life Sci..

[33] Choi J.W., Ohn J.H., Jung H.S., Park Y.J., Jang H.C., Chung S.S., Park K.S. (2018). Carnitine induces autophagy and restores high-fat diet-induced mitochondrial dysfunction. Metabolism.

[34] Astashkin I., Glazer M.G. (2016). The effect of L-carnitine on oxidative stress in cardiovascular diseases. Med. Counc..

[35] Poljsak B., Raspor P. (2008). The antioxidant and pro-oxidant activity of vitamin C and trolox in vitro: A comparative study. Appl. Toxicol..

[36] Giordano M.E., Caricato R., Lionetto M.G. (2020). Concentration Dependence of the Antioxidant and Prooxidant Activity of Trolox in HeLa Cells: Involvement in the Induction of Apoptotic Volume Decrease. Antioxidants.

[37] Tafazoli S., Wright J.S., O’Brien P.J. (2005). Prooxidant and antioxidant activity of vitamin E analogues and troglitazone. Chem. Res. Toxicol..

[38] Ahmad S.U., Bhat A.M., Ragni G., Mushtaq M.A., Abdullah S.T. (2025). Antioxidant-induced autophagy ameliorates oxidative mediated endoplasmic reticulum stress in ultraviolet-B-exposed human primary dermal fibroblasts. Exp. Gerontol..

[39] Nobelos P.T., Kourounakis P.N., Rekka E.A. (2017). Anti-inflammatory and Hypolipidemic Effect of Novel Conjugates with Trolox and Other Antioxidant Acids. Med. Chem..

[40] Sacks B., Onal H., Martorana R., Sehgal A., Harvey A., Wastella C., Ahmad H., Ross E., Pjetergjoka A., Prasad S. (2021). Mitochondrial targeted antioxidants, mitoquinone and SKQ1, not vitamin C, miti-gate doxorubicin-induced damage in H9c2 myoblast: Pretreatment vs. co-treatment. BMC Pharmacol. Toxicol..

[41] Nahar N., Sohag M.S.U. (2025). Advancements in mitochondrial-targeted antioxidants: Organelle-specific drug delivery for disease management. Adv. Redox Res..

[42] Plotnikov E.Y., Zorov D.B. (2019). Pros and Cons of Use of Mitochondria-Targeted Antioxidants. Antioxidants.

[43] Maekawa S., Takada S., Nambu H., Furihata T., Kakutani N., Setoyama D., Ueyanagi Y., Kang D., Sabe H., Kinugawa S. (2019). Linoleic acid improves assembly of the CII subunit and CIII2/CIV complex of the mitochondrial oxidative phosphorylation system in heart failure. Cell Commun. Signal..

[44] Jang S., Javadov S. (2023). Unraveling the mechanisms of cardiolipin function: The role of oxidative polymerization of unsaturat-ed acyl chains. Redox Biol..

[45] Zhigacheva V.I., Krikunova I.N., Binyukov I.V., Mil E., Rusina I., Goloshchapov A. (2023). Ethoxidol as a broad-spectrum adaptogen. Curr. Mol. Pharmacol..

[46] Council of Europe (1986). European Convention for the Protection of Vertebrate Animals Used for Experimental and Other Scientific Purposes (ETS No. 123).

[47] Golovina R.V., Kuzmenko T.E. (1977). Thermodynamic evaluation interaction of fatty acid methyl esters with polar and nonpolar stationary phases, based on their retention indices chromatographia. Chromatography.

[48] Karkishchenko V.N., Karkishchenko N.N., Shustov E.B., Berzin I.A., Fokin Y.V., Alimkina O.V. (2016). Features of interpretation of performance indicators of laboratory animals according to swimming tests with load. Biomedicine.

