# Supplementary material for: Biological Activity of 3-Hydroxypyridine Derivatives with Natural Components
Source: Int J Mol Sci. 2026 Oct 7;27(19):8903. doi: 10.3390/ijms27198903 (PMC13665236; doi:10.3390/ijms27198903)
Supplement: Supplementary file 1 [file ijms-27-08903-s001.zip › ijms-4551518-supplementary.pdf]

Supplementary Materials:

Model of acute hypobaric hypoxia

Acute hypobaric hypoxia was induced in mice using a hyperbaric chamber set to a low-pressure environment of 230.40 mm Hg, simulating an altitude of 9,000 meters. The experiment began by establishing a pressure equivalent to 5,000 meters (405 mm Hg). Subsequently, the simulated altitude was increased by 1,000 meters every minute until reaching the target of 9,000 meters, at which point the subjects were maintained for a duration of 5 minutes.

Acute hemic hypoxia model

To induce acute hemic hypoxia, Balb/c mice were injected intraperitoneally with sodium nitrite at a dose of 250 mg/kg.

Acute cytotoxic hypoxia model

An intraperitoneal injection of sodium azide (20 mg/kg) was used to model acute cytotoxic hypoxia in mice.

Dynamic performance model

Physical performance was measured using a forced swim test, where mice swam in room-temperature water while bearing a load of 2% of their body weight until they reached the point of fatigue.

Table S1. Protective Activity of Carnitinate 2-Ethyl-6-methyl-3-hydroxypyridine carnitinate 2-Ethyl-6-methyl-3-hydroxypyridine (Results of 10 Technical and 6 Bbiological Replicates are Presented).

Impact	Measured parameter	Control	CAR-3-HP $2{\times}10^{-9}$mol/kg
Ascent to an altitude of 11.000 meters (210 mm Hg) (*hypobaric hypoxia*)	Life expectancy in minutes	4.0±1.2	13.9±1.5
	% of survivors	11%	35%
Sodium nitrite injection 250 mg/kg (*hemic hypoxia*)	Life expectancy in minutes	4.5±1.7	15.2±2.0
	% of survivors	14%	26%
Sodium azide injection 20 mg/kg (*cytotoxic hypoxia*)	Life expectancy in minutes	2.4±0.5	13.3±1.0
	% of survivors	0%	35%
Swimming with a load at room temperature	Life expectancy in minutes	4.5±0.5	15.8±1.1
	% of survivors	100%	100%

Table S2. Protective Activity of Carboxychromanate of 2-Ethyl-6-methyl-3-hydroxypyridine (Results of 10 Technical and 6 Bbiological Replicates are Presented).

Impact	Measured parameter	Control	CAR-3-HP $2{\times}10^{-9}$mol/kg
Ascent to an altitude of 11.000 meters (210 mm Hg) (*hypobaric hypoxia*)	Life expectancy in minutes	3.9±1.4	7.4±3.0
	% of survivors	10%	20%
Sodium nitrite injection 250 mg/kg (*hemic hypoxia*)	Life expectancy in minutes	3.5±1.2	5.2±1.4
	% of survivors	14%	23%
Sodium azide injection 20 mg/kg (*cytotoxic hypoxia*)	Life expectancy in minutes	2.5±0.7	4.3±1.4
	% of survivors	0%	22%

B: Detection of LC_3B western blotting in columns (1 and, 2– starvation, ,3 and 4 - CAR-3-HP , 5 and 6 control, 7- protein marker)

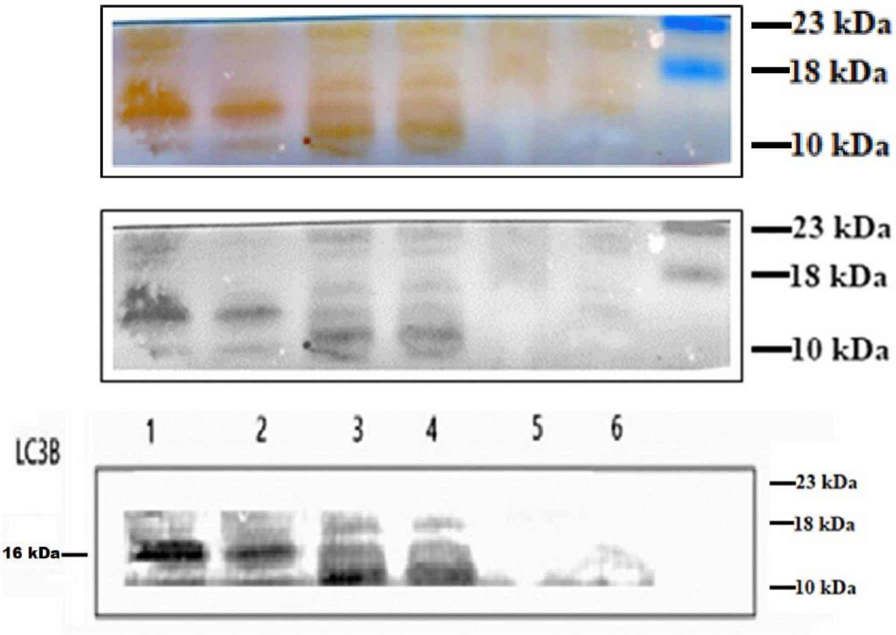

C: Detection of Beclin1 western blotting in columns (1 and 2 - starvation, 3 and 4 – CAR-3-H, 5 and 6 – control, 7 - protein marker)

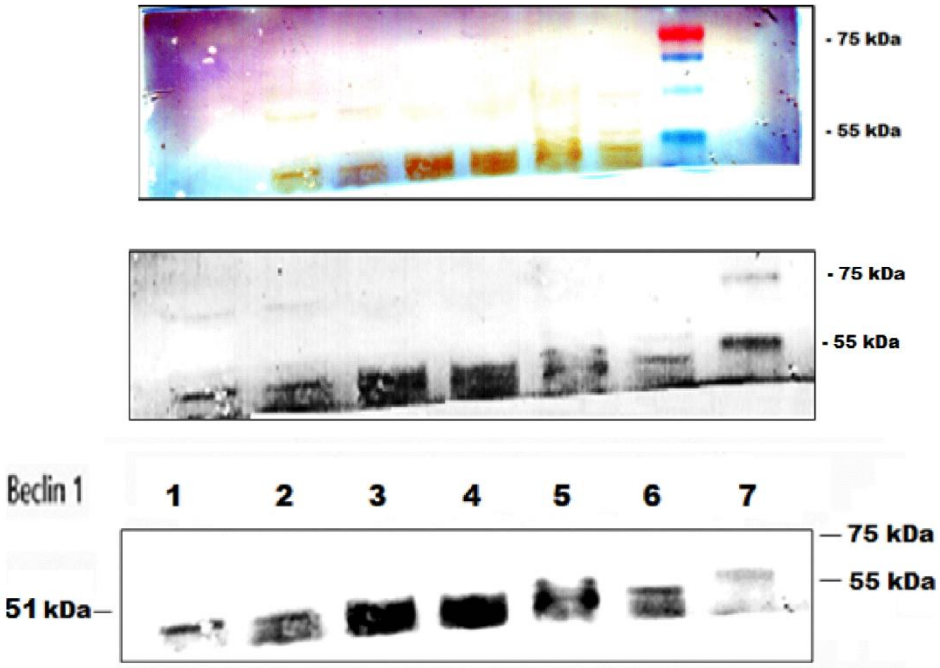

. **B:** Detection of LC3B western blotting in columns (1 and, 2– starvation, ,3 and 4 -CAR-3-HP , 5 and 6 control, 7- protein marker); **C:** Detection of Beclin1 western blotting in columns (1 and 2 - starvation, 3 and 4 – CAR-3-H, 5 and 6 – control, 7 - protein marker); **D:** Detection of LC3B western blotting in columns (1 - control, 2 - CAR-3-H, 3 - CCR-3-HP, 4 and 5 – control, 6 - protein marker)

D: Detection of LC_3B western blotting in columns (1 - control, 2 - CAR-3-H, 3 - CCR-3-HP, 4 and 5 – control, 6 - protein marker)

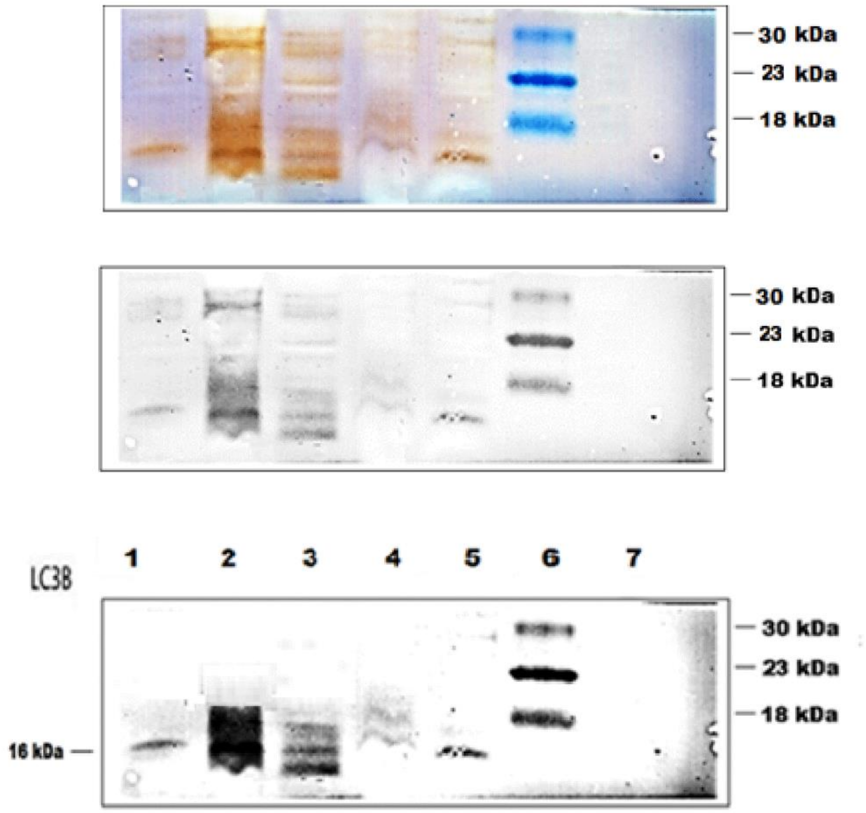

Figure S1F. **B:** Detection of LC3B western blotting in columns (1 and, 2– starvation, ,3 and 4 -CAR-3-HP , 5 and 6 control, 7- protein marker); **C:** Detection of Beclin1 western blotting in columns (1 and 2 - starvation, 3 and 4 – CAR-3-H, 5 and 6 – control, 7 - protein marker); **D:** Detection of LC3B western blotting in columns (1 - control, 2 - CAR-3-H, 3 - CCR-3-HP, 4 and 5 – control, 6 - protein marker)
